# Ascorbic acid promotes 3T3-L1 cells adipogenesis by attenuating ERK signaling to upregulate the collagen VI

**DOI:** 10.1186/s12986-017-0234-y

**Published:** 2017-12-28

**Authors:** Chuanguo Liu, Kun Huang, Guorong Li, Pingping Wang, Chang Liu, Congcong Guo, Zongguo Sun, Jie Pan

**Affiliations:** Shandong Provincial Key Laboratory of Animal Resistant Biology, College of Life Sciences, Shandong Normal University, Jinan, 250014 China

**Keywords:** Ascorbic acid, 3T3-L1 cells, Collagen VI, ERK signaling, Adipogenesis

## Abstract

**Background:**

Type VI collagen is supposed to be a regulation factor in adipogenesis. This study aimed to assess the promoting effect of vitamin C (VC) on adipogenic differentiation of preadipocytes as well as its mechanism.

**Methods:**

Five sets of different combinations of chemicals were used to inhibit synthesis of type I to VI collagens, blocking ERK1/2 phosphorylation during adipogenesis of 3T3-L1 preadipocytes. Furthermore, to explore whether collagen VI plays a critical role during adipogenesis, specific knockdown of collagen VI was performed by using RNA interference. The morphology and expression patterns of several target factors involved in adipogenesis were assessed at various time points.

**Results:**

A reduction in ERK1/2 phosphorylation and an increase in collagen VI and adipogenic-specific factors, such as C/EBPβ, PPARγ and C/EBPα, were observed after treating adipogenic 3T3-L1 cells with AA2P, a stable derivative of VC. Inhibition of collagen synthesis by ethyl-3, 4-dihydroxybenzoate (EDHB) or by specific knockdown of collagen VI by RNAi could promote ERK1/2 phosphorylation. The ERK1/2 phosphorylation in both cases could be attenuated by AA2P treatment. In addition, the inhibition of ERK1/2 phosphorylation by U0126, a highly selective inhibitor of both MEK1 and MEK2 and a type of MAPK/ERK kinase, up-regulated the expression of collagen VI, while it down-regulated the adipogenic-specific factors.

**Conclusion:**

AA2P could up-regulate the expression of collagen VI by attenuating ERK1/2 phosphorylation, further up-regulating adipocyte-specific factors, thus finally promoting the adipogenesis of 3T3-L1 preadipocytes.

**Electronic supplementary material:**

The online version of this article (10.1186/s12986-017-0234-y) contains supplementary material, which is available to authorized users.

## Background

In addition to significant increases in protein expression directly related to lipid metabolism, the type and level of ECM components secreted during adipogenesis also change [1, 2]. It has been reported that the progression from preadipocytes to mature adipocytes is required for remodeling of the ECM [3]. Collagens in the ECM are secreted with the differentiation of adipocytes, and each type of collagen secretes at a specific stage of adipogenesis. Collagens I and III are secreted in the early stage of adipogenesis to construct the cell scaffolds. Basal membrane components, such as collagen IV, reached a peak in the middle stage of adipogenesis. Microfiber collagen components such as collagen V and VI secretion also reached a maximum level during adipogenesis [4]. This remodeling, which encompasses increased flexibility of ECM and changes in the connection among collagens, can promote the transcription of adipocyte-specific genes, such as CCAAT/enhancer-binding protein β (C/EBPβ) and peroxisome proliferator-activated receptor γ (PPARγ) [5].

Collagen VI is comprises the three subunits of α1, α2 and α3, which are encoded by different genes. The silencing of the Col6α1 gene can prevent the secretion and assembly of collagen VI in the ECM, affecting its function [6–8]. Compared to other types of collagen, collagen VI is enriched in adipocytes and is associated with adipogenesis and obesity-related diseases [1, 2, 9, 10]. However, the roles played by collagen VI in regulating adipogenesis or adipocyte hypertrophy have not been fully addressed.

Ethyl-3, 4-dihydroxybenzoate (EDHB) is a known ideal inhibitor through which we can explore the role of collagens in regulating adipogenesis of preadipocytes [1]. It inhibits the activity of prolyl-4-hydroxylase, resulting in a non-functional degradable protein and the failure of collagen triple helix formation and preventing the secretion and assembly of type I-VI collagens [11]. It has been reported that collagen V and VI play a primary role in the adipogenesis of the bovine intramuscular preadipocyte cell line (BIP). The triglyceride (TG) content of BIP is reduced by 50% after EDHB treatment. Moreover, the adipogenic rate restores 23.2% and 31.5% of the cells cultured on dishes coated with collagen V and VI, respectively. There was no significant effect of BIP on TG accumulation, which was cultured on dishes coated with other types of collagen [1]. These data indicated that collagen VI plays a primary role during adipogenesis, while lacking a discussion of its mechanism.

In addition to its antioxidant activity, vitamin C (VC) acts as a cofactor of the hydroxylating enzyme of proline and lysine residues in procollagen, which stabilizes the collagen triple helix. Moreover, VC regulates collagen synthesis, cell growth, and cell differentiation [12, 13]. It has been reported that VC can enhance stem cells to differentiate into cardiac lineages by enhancing collagen synthesis [14–16], and it can enhance neurons by increasing the expression of genes involved in neurogenesis [17]. In addition, AA2P, a stable derivative of VC that possesses similar biological properties has been demonstrated to stimulate lipid accumulation by increasing the collagen IV synthesis [18] and through differential expression of collagens [19]. Moreover, AA2P also promotes adipogenesis of BMSCs by increasing the accumulation of collagens, but it does not specify the type of collagen [20, 21]. Therefore, the question remains as to whether differential expression of collagens and the accumulation of collagen stimulate lipid accumulation.

Extracellular signal-regulated kinase (ERK) is a member of the MAPK family, which controls cell proliferation, differentiation and stress response [22]. The ERK signal plays an important role in adipogenesis; however, its specific effect is divergent. In fact, several studies suggest that ERK1/2 could promote adipogenesis and U0126, which is a specific inhibitor of MEK1 and MEK2; thus, ERK inactivation could inhibit adipogenesis [23–25]. While others propose an inhibitory role for ERK1/2 during adipogenesis [26, 27].

There is also a link between VC and ERK. It has been reported that AA2P promotes the production of ECM components by attenuating the phosphorylation of ERK1/2 and enhances the stemness of adipose-derived stem cells and the cell sheet formation, while it has no effect on the subsequent adipogenic differentiation [28]. In other studies, VC attenuates the phosphorylation of ERK1/2 to inhibit the regulation of type I and III collagen production by the LL-37 polypeptide in human dermal fibroblasts [29]. Based on the above evidence, we hypothesized that AA2P attenuates the phosphorylation of ERK1/2 to up-regulate the expression of collagen VI and promote the adipogenesis of 3T3-L1 cells.

## Methods

### Chemicals

AA2P, EDHB and U0126 were purchased from Sigma-Aldrich (Milwaukee, Wisc., USA). AA2P was initially dissolved in Dulbecco’s modified Eagle’s medium (DMEM, Hyclone) in a 250 mM. EDHB was initially dissolved in absolute ethanol in a 100 mM. U0126 was initially dissolved in dimethyl sulfoxidein (DMSO) in a 100 mM. The final ethanol and DMSO concentration of the controls were adjusted to 0.1%.

### Adipocyte differentiation and chemical treatment

**Fig. 1:**
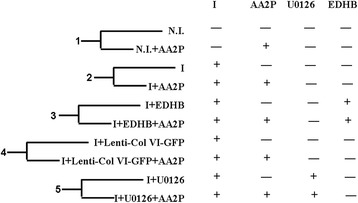
The experimental groups. Group I represents the adipogenic group, when the cells reached 100% confluence after 2 days (set for 0 days, D0), and the replacement of the adipogenic inducers (based on the growth medium, insulin, dexamethasone and 3-isobutyl-1-methylxanthine) was added to induce the adipogenic differentiation of 3 T3-L1 preadipocytes for 2 days, after 14 days (D14) of continuous induction. In addition, the I + AA2P group, the I + EDHB group the I + U0126 group were added to AA2P, EDHB and U0126 based on the I group at D0, and were continuously induced for 14 days (D14). N.I. represents the non-induced group; the Lenti-Col VI-GFP represents the Col VI knockdown group that used RNAi

To produce mature adipocytes, 3T3-L1 preadipocytes (American Type Culture Collection, Manassas, VA) were seeded on 12-well plates with growth medium to let the cells reach 100% confluence. Two days later, the cells were induced to become mature adipocytes with a cocktail adipogenic inducers, defined as day 0 (D0) for 2 days. Next, the cells were refreshed with the maintenance medium for an additional 2 days. The medium was finally replaced with growth medium for 10 more days. The cells were treated with relative chemicals from days 0 to 14 as follows: the non-induction group (N.I.); the AA2P alone treated group (N.I. + AA2P); the adipogenic group (I), 250 μM AA2P group (I + AA2P); the 100 μM EDHB group (I + EDHB); the 100 μM EDHB plus 250 μM AA2P group (I + EDHB + AA2P); the RNAi groups; the 100 μM U0126 group (I + U0126); and the 100 μM U0126 plus 250 μM AA2P group (I + U0126 + AA2P). The experimental groups were shown in Fig. 1.

### Construction of lentiviral vector for silencing of mouse collagen VI α1 subunit expression

Small hairpin RNA (shRNA) of the mouse collagen VI α1 subunit lentivirus gene transfer vector encoding the GFP sequence was designed. Different siRNAs were screened by co-transfection with mouse collagen VI α1 subunit cDNA plasmid into HEK293T cells with Lipofectamine 2000 (Invitrogen Corporation, Carlsbad, CA, USA). The optimal sequence of the siRNA against the mouse collagen VI α1 subunit (5’-ACCTATTCCGTGTACCAAA-3′) (GenBank gi: NM_009933), was then cloned into the plasmid GV 112, which encodes an HIV-derived lentiviral vector containing a multiple cloning site for insertion of the shRNA constructs to be driven by an upstream U6 promoter and a downstream cytomegalovirus promoter with GFP. The resulting lentiviral vector containing mouse collagen VI α1 subunit shRNA was named Lenti-ColVI-GFP, and its sequence was confirmed by PCR and sequencing analysis. A negative control (NC) lentiviral vector (Lenti-GFP) containing NC shRNA was constructed by a similar process (NC lentivirus, 5’-TTCTCCGAACGTGTCACGT-3′).

### Lentivirus siRNA gene transfection

3T3-L1 preadipocytes were plated in 12-well plates overnight and infected with Lenti-ColVI-GFP by addition of lentivirus diluted into 0.5 ml of Dulbecco’s modified eagle medium (DMEM) containing 5% NBCS and polybrene at an MOI of approximately 100 for 12 h at 37 °C. The medium was then replaced with growth medium until it reached 100% confluence. Adipogenic induction was initiated after 48 h of confluence. The controls were infected with NC lentivirus (I + Lenti-GFP).

### Oil red O staining

The cells were stained with Oil Red O (Sigma-Aldrich) at the D3, D7 and D14 after induction for determining the degree of differentiation (the ratio of adipose cells). The lipid staining was quantified by extracting the dye with 100% isopropanol and measuring the absorbance at 520 nm.

### RNA isolation and real-time quantitative reverse transcription (RT)-PCR

Total RNA was extracted from 3T3-L1 cells at the D0, D3, D7 and D14 after induction using AxyPrep Total RNA Preparation Kit (Axygen, American) according to the manufacturer’s protocol. RNA concentration was determined by optical density at 260 nm (OD 260) using Nanodrop (Thermo Scientific, MA, USA). Total RNA (0.3 μg) was reverse-transcribed using First Strand cDNA Synthesis Kit (TOYOBO, FSK-100). The expression level of genes was assessed by quantitative RT-PCR. cDNA product (0.2 μg) was amplified by quantitative PCR with SuperReal RT-PCR PreMix (SYBR Green) kit (Tiangen, China, FP204). The results are expressed as the fold increase relative to the controls after normalizing to the β-actin gene expression levels. The sequences of the gene-specific primers used are shown in Table 1.

**Table 1:** The sequences of the gene-specific primers

Gene	GenBank No.	Primer	Length
Collagen VI α1	NM_009933	F: 5’**-**TGCCCTGTGGATCTATTCTTCG**-**3′ R: 5’**-**CTGTCTCTCAGGTTGTCAATG**-**3′	107 bp
SREBP-1c	NM_011480	F: 5’**-**GTGAGCCTGACAAGCAATCA**-**3′ R: 5’**-**GGTGCCTACAGAGCAAGAG**-**3’	102 bp
C/EBPβ	NM_009883	F: 5’**-**ACACGGGACTGACGCAACAC**-**3′ R: 5’**-**AACCCCGCAGGAACATCTTT**-**3’	78 bp
PPARγ2	NM_011146	F: 5’**-**GTCATCCTGCTCTTCTTTCTCG**-**3′ R: 5’**-**ATGGCGTCCCTTCTCCTGT**-**3’	115 bp
C/EBPα	NM_007678	F: 5’**-**CAGGAGGAAGATACAGGAAGC**-**3′ R: 5’**-**TCTCCATGAACTCACCCAGG**-**3’	229 bp
β-actin	NM_007393	F: 5’**-**CCTCTATGCCAACACAGTGC**-**3′ R: 5’**-**GTACTCCTGCTTGCTGATCC**-**3’	193 bp

*F* Forward primer, *R* Reverse primer

### Western blotting

Total cell proteins were isolated using a lysis buffer and sonicated as previously reported [30]. An aliquot containing 30 μg of proteins was separated by electrophoresis on a 12% sodium dodecyl sulfate-polyacrylamide gel electrophoresis (SDS-PAGE) and was electrophoretically transferred to PVDF membranes (Millipore, Massachusetts, USA). The membranes were blocked in 10 mM of Tris buffer solution (TBS, pH 7.5) containing 0.1% Tween 20 (TBS-T) and 5% BSA (Beyotime, Shanghai, China) for 1 h at room temperature. After washing with TBS-T, the membranes were incubated with primary antibodies (anti-collagen VI α1 (Abcam, Cambridge, MA), anti-ERK1/2, anti-phospho-ERK1/2, anti-PPARγ, anti-C/EBPα (all from Cell Signalling, Danvers, MA), anti-SREBP-1c and anti-C/EBPβ (all from Santa Cruz, California, USA) overnight at 4 °C. After extensive washing, the membranes were further incubated with the corresponding secondary antibodies (horseradish peroxidase-conjugated goat anti-mouse and goat anti-rabbit IgG, Jackson, PA, USA) for 1.5 h at a dilution of 1:10,000. The proteins were detected by the Immobilon Western Chemiluminescent HRP Substrate (Millipore, MA, USA) and were displayed on X-ray film (Fujifilm, Tokyo, Japan). QuantiScan 3.0 was used to analyze the gray value of the protein blot. The gray value of the target protein blot divided by the gray value of the internal reference GAPDH and/or α-tubulin (GenScript, Piscataway, NJ, USA) represents the relative expression of the target protein.

### Statistical analysis

Data were analyzed using SPSS version 19.0 software (SPSS Inc., Chicago, IL, USA) and two tailed in tests. Each experiment was performed in at least triplicate. The data are expressed as the mean ± SEM with GraphPad Prism 5. Differences between groups were analyzed by conducting one-way analysis of variance (ANOVA) followed by Student’s t test. *P < 0.05 was considered statistically significant and significant between groups.

## Results

### AA2P promotes adipogenesis of 3T3-L1 cells

**Fig. 2:**
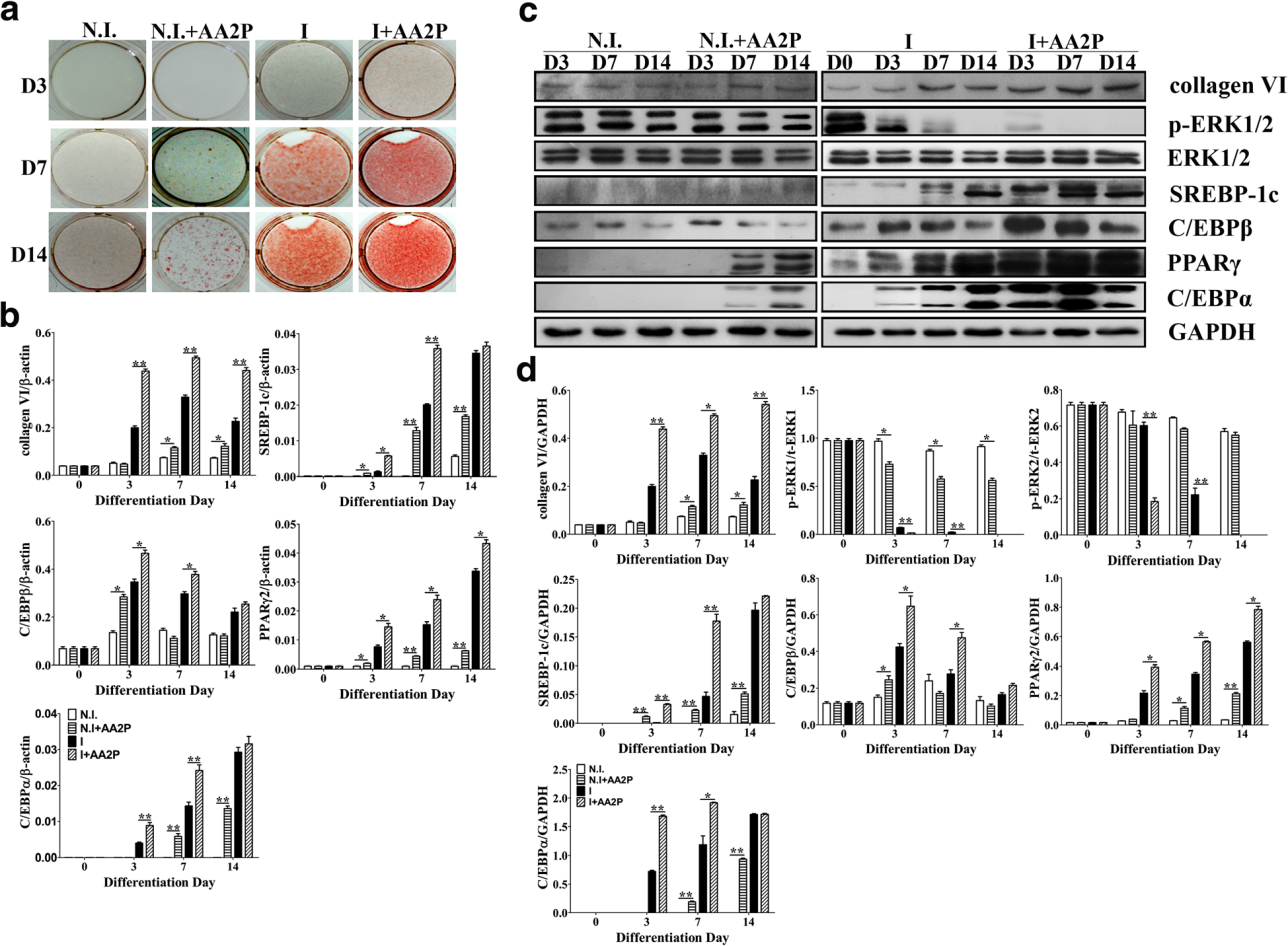
AA2P promotes the adipogenesis of 3T3-L1 cells. Oil red O staining, RNA and protein extraction were performed at D0, D3, D7 and D14 after non-induction or induction. AA2P was added to induce the 3T3-L1 preadipocytes on the overall stage of adipogenesis. **a** Oil red O staining; **b** mRNA levels of collagen VI and adipogenic-specific genes in the 3T3-L1 cells detected by qPCR. mRNA levels of the target gene are normalized to β-actin. Values are expressed as the mean ± SEM. *P < 0.05, **P < 0.01 versus the control group (*n* = 3); **c** Western blotting results at the same stage with mRNA levels; **d** Relative expression of the target protein

Consistent with a previous study [18], AA2P alone could induce the adipogenesis of 3T3-L1 mouse preadipocytes without adipogenic induction, although this effect was weaker. On the other hand, lipid droplets began to be accumulate from D3 when the cells were treated with adipogenic inducer. With the progress of adipogenesis, lipid droplets gradually increased and reached a peak at D7. Compared with this group, more evident adipogenesis was found in the I + AA2P group (Fig. 2a).

Further studies had shown that AA2P significantly up-regulated the expression of collagen VI, SREBP-1c, C/EBPβ, PPARγ2 and C/EBPα (*P* < 0.05) in both the non-induction group and the adipogenic group. However, their expression in the late stage (D8-D14) tended to be consistent, except for PPARγ2 in the adipogenic group (Fig. 2b–d). The total ERK1/2 remained essentially unchanged; however, the ERK1/2 phosphorylation (*P* < 0.05) was gradually reduced, and there was almost no expression in the late stage of adipogenesis. In the early and middle stages of adipogenesis, ERK1/2 phosphorylation (*P* < 0.05) in the AA2P treatment group was significantly lower than that in the adipogenic group. These data suggest that AA2P may attenuate the ERK1/2 phosphorylation to up-regulate the expression of collagen VI and further up-regulate the expression of SREBP-1c, C/EBPβ, PPARγ2 and C/EBPα during the adipogenesis of 3T3-L1 cells. The further question is raised: is the above mechanism by which AA2P promotes the adipogenesis of 3T3-L1 cells correct?

### Inhibition of collagens affects the adipogenesis of 3T3-L1 cells

**Fig. 3:**
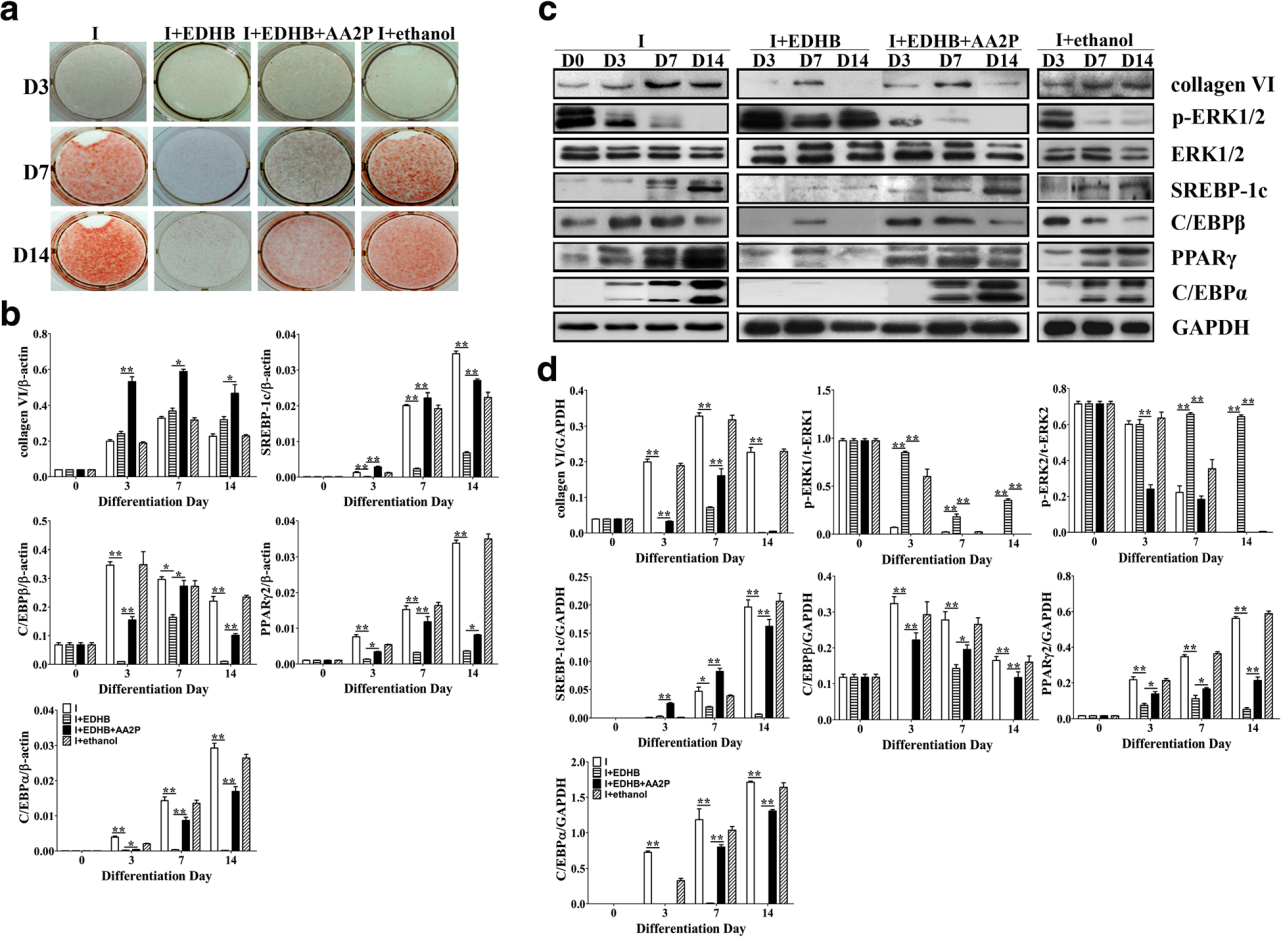
Inhibition of collagen affects the adipogenesis of the 3T3-L1 cells. Oil red O staining, RNA and protein extractions were performed at D0, D3, D7 and D14 after induction. EDHB was added at the overall stage of adipogenesis or after treating with AA2P. **a** Oil red O staining; **b** mRNA levels of collagen VI and adipogenic-specific genes in the 3T3-L1 cells detected by qPCR. mRNA levels of the target gene are normalized to β-actin. Values are expressed as the mean ± SEM. *P < 0.05, **P < 0.01 versus the control group (*n* = 3); **c** Western blotting results at the same stage with mRNA levels; **d** Relative expression of the target protein

To confirm the effect of collagens on adipogenesis, 100 μM of EDHB was added on the overall stage of adipogenesis or after treatment with AA2P. The results showed that EDHB completely inhibited the adipogenesis of 3T3-L1 cells (Fig. 3a, I+EDHB group), indicating that collagens played an important role in the process. Notably, the inhibitory effect of EDHB on cell adipogenesis was partially reversed by AA2P (Fig. 3a, I+EDHB + AA2P group).

It has been reported that the failure of the hydroxylation of collagen subunits will result in a non-functional degradable protein [11]. We examined the expression of SREBP-1c, C/EBPβ, PPARγ2 and C/EBPα. The results showed that EDHB significantly reduced the assembly and secretion of collagen VI and inhibited the expression of SREBP-1c, C/EBPβ, PPARγ2 and C/EBPα (*P* < 0.01) (Fig. 3b–d). In contrast, the expression of collagen VI was significantly up-regulated at the transcriptional level (Fig. 3b, I+EDHB group). The possible reason for this is that EDHB inhibited the assembly of collagen subunits without inhibiting the transcription level, and collagen VI was up-regulated by positive feedback after the synthesis of the collagen VI was inhibited.

Moreover, our results showed that EDHB could significantly increase the ERK1/2 phosphorylation (*P* < 0.01), and the expression trend was opposite with collagen VI (Fig. 3c and d). This finding suggested that there may be interactions between the two molecules. Additionally, ERK1/2 phosphorylation (*P* < 0.01) was significantly decreased after treating with AA2P, indicating that AA2P could attenuate ERK phosphorylation. These data demonstrate that AA2P may attenuate ERK phosphorylation to up-regulate the expression of collagen VI during the adipogenesis of 3T3-L1 cells. The data shows that adipogenesis was inhibited when the expression of type I to VI collagen was suppressed, and it was reversed partially when treated with AA2P, indicating that collagens play a key role during adipogenesis. Another question is raised: is collagen VI among the types of collagen that have more importance?

### Knockdown of collagen VI inhibits the adipogenesis of 3T3-L1 preadipocytes

**Fig. 4:**
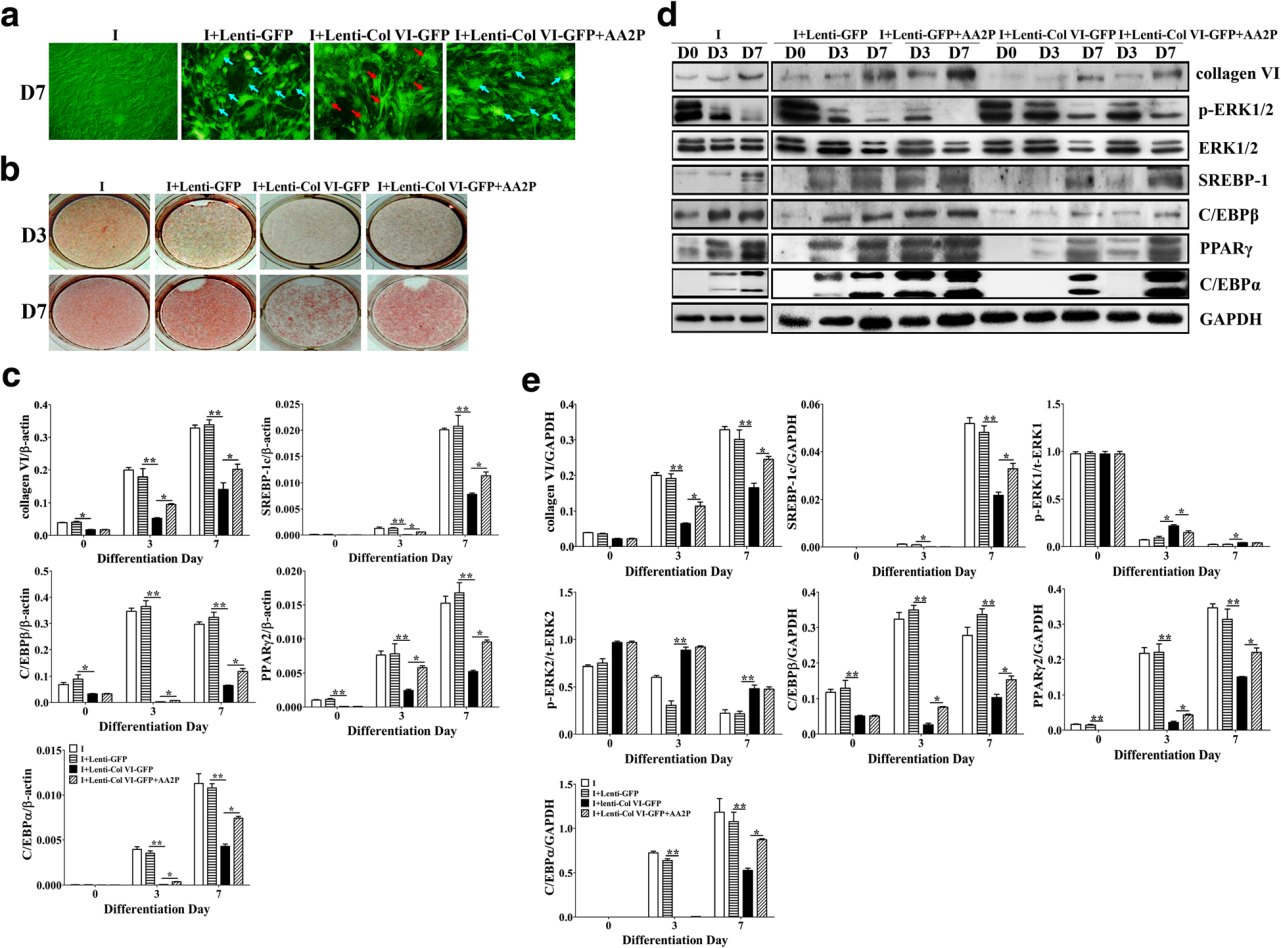
Knocking down collagen VI inhibits the adipogenesis of 3T3-L1 cells. The fluorescence abundance reached a peak after 72 h of transfection, and the adipogenic inducers were added. Oil red O staining, RNA and protein extractions were performed at D0, D3 and D7 after induction. **a** The transfection results of the 3T3-L1 with Lenti-GFP and Lenti-ColVI-GFP (MOI = 100). The blue arrows point to the cells with lipid droplets, while the red arrows point to the un-adipogenic cells by Lenti-ColVI-GFP; **b** Oil red O staining; **c** mRNA levels of collagen VI and adipogenic-specific genes in the 3T3-L1 cells detected by qPCR. mRNA levels of the target gene are normalized to β-actin. Values are expressed as the mean ± SEM. *P < 0.05, **P < 0.01 versus the control group (*n* = 3); **d** Western blotting results at the same stage with mRNA levels. **e** Relative expression of the target protein

To further verify the role of collagen VI during adipogenesis, collagen VI knockdown was performed by using RNAi. The results showed that knockdown of collagen VI significantly inhibited the adipogenesis of 3T3-L1 cells (Fig. 4a and b) due to inhibited the expression of SREBP-1c, C/EBPβ, PPARγ2 and C/EBPα (*P* < 0.01) (Fig. 4c–e). The knockdown efficiency of collagen VI was approximately 65.3% (see Additional file 1), and the ratio of adipose cells was reduced by approximately 51.6%. The ratio was partially restored after treatment with AA2P, indicating that AA2P promoted the synthesis of collagen VI, which corroborated previous reports.

We further detected the phosphorylation of ERK1/2. The results showed that knockdown of collagen VI can significantly promote the phosphorylation of ERK1/2, especially ERK1 phosphorylation (*P* < 0.05); therefore, the expression of PPARγ2 in the Lenti-ColVI-GFP group was significantly lower (*P* < 0.01) than that in the control group. The ERK1 phosphorylation was significantly down-regulated (*P* < 0.05) after treatment with AA2P at D3, further demonstrating that AA2P could attenuate ERK1/2 phosphorylation, especially the ERK1 phosphorylation (Fig. 4d and e). These data further demonstrate the interaction between collagen VI and ERK signaling and that AA2P attenuates the ERK1/2 phosphorylation to up-regulate the expression of collagen VI during the adipogenesis of 3T3-L1 cells.

### Inhibiting the ERK1/2 phosphorylation affects the adipogenesis of 3T3-L1 cells

**Fig. 5:**
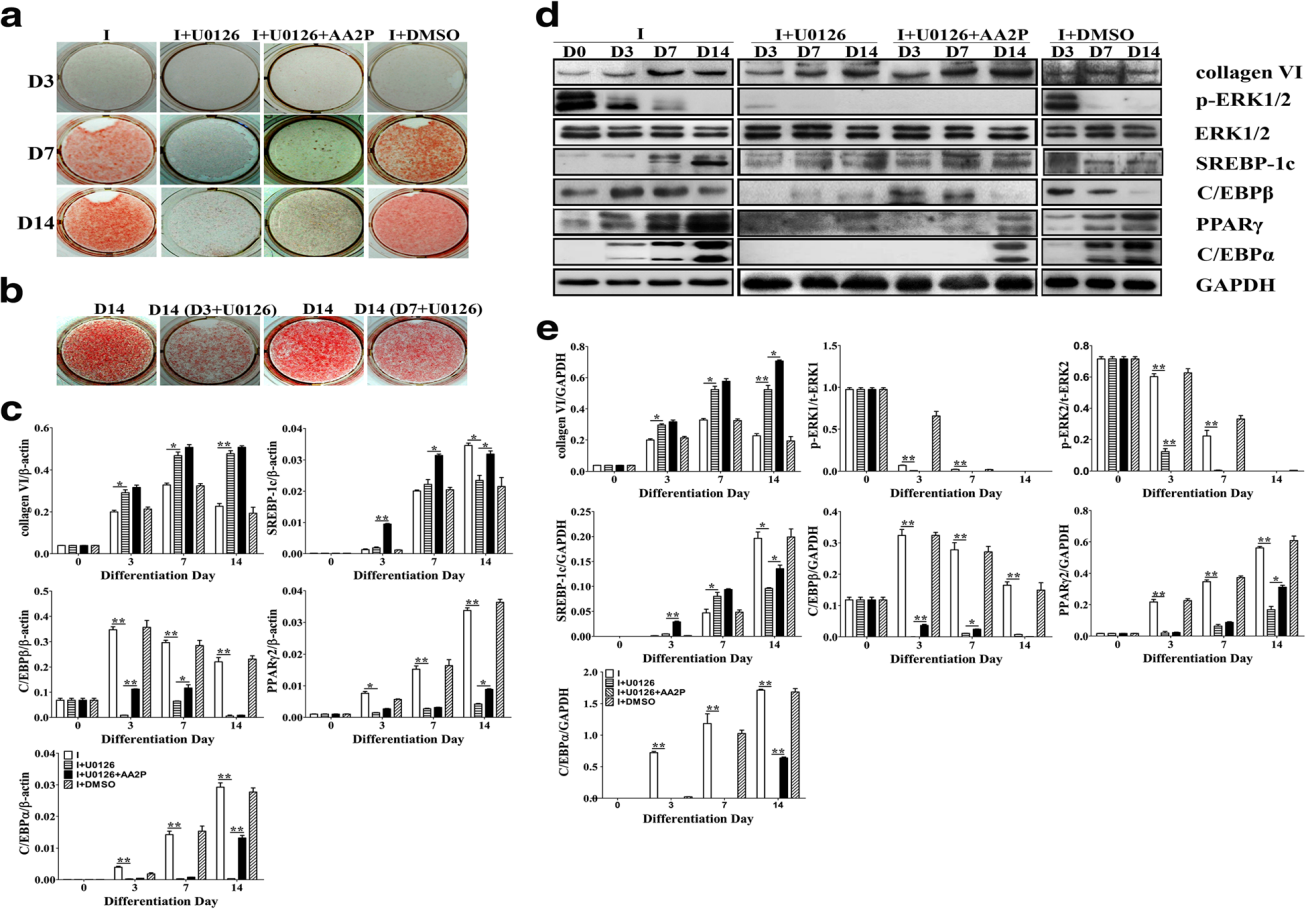
Inhibiting ERK1/2 phosphorylation affects the adipogenesis of 3T3-L1 cells. Oil red O staining, RNA and protein extraction were performed at D0, D3, D7 and D14 after induction. U0126 (100 μM), a specific inhibitor of ERK1/2 phosphorylation, was added at the overall phase, after treatment with 250 μM of AA2P (from D0 to D14), or at the early (form D3 to D14) or middle (form D7 to D14) stage of adipogenesis. **a** Oil red O staining; **b** The effect of U0126 on adipogenesis was added at the early (D3) and middle stage (D7), Oil red O staining was performed at D14 after induction; **c** mRNA levels of collagen VI and adipogenic-specific genes in the 3T3-L1 cells detected by qPCR. mRNA levels of the target gene are normalized to β-actin. Values are expressed as the mean ± SEM. *P < 0.05, **P < 0.01 versus the control group (*n* = 3); **d** Western blotting results at the same stage with mRNA levels; **e** Relative expression of the target protein

To further verify the effect of ERK signaling on adipogenesis, a specific inhibitor of MEK1 and MEK2, U0126, which also activated ERK (ERK1/2 phosphorylation), was added at the overall stage of adipogenesis or after treatment with AA2P. U0126 completely inhibited the adipogenesis of 3T3-L1 cells (Fig. 5a) and inhibited the expression of C/EBPβ, PPARγ and C/EBPα (*P* < 0.01) (Fig. 5c–e). Furthermore, we added U0126 at D3 and D7. The results showed that ERK1/2 phosphorylation mainly regulated the adipogenesis of 3T3-L1 cells in the early and middle stage of adipogenesis (Fig. 5b).

The above results appear to be contrary to our expected results. However, previous studies have shown that ERK1/2 phosphorylation in the early stage of adipogenesis is essential for C/EBPs and PPARγ transcription, while PPARγ is a downstream ERK1/2 molecule and the activation of ERK1/2 could phosphorylate PPARγ, leading to a reduction in its transcriptional activity, thereby inhibiting adipogenesis [31]. Moreover, our data suggest that the lack of ERK signaling could not transmit the collagen signal into the cells.

Collagen VI significantly increased (*P* < 0.05) after U0126 treatment, further indicating that there is an interaction between collagen VI and ERK signaling. Moreover, AA2P could regulate the expression of collagen through ERK signaling (Fig. 5c–e). Surprisingly, the expression of SREBP-1c was not inhibited by U0126 in the early and middle stage of adipogenesis. However, it was inhibited in the late stage of adipogenesis. This may be suggested the fact that the ERK1/2 phosphorylation inhibits the expression of SREBP-1c. These data further demonstrate that AA2P could attenuate ERK1/2 phosphorylation to up-regulate the expression of collagen VI, and that ERK signaling is necessary to transmit the collagen signal into the cells.

## Discussion

It is known that VC is not only an important antioxidant also an indispensable nutrient. Recently, with the development of induced pluripotent stem cell technology, VC has been used to improve the efficiency of induced pluripotent stem cells [32]. Studies have shown that VC can regulate the adipogenesis of preadipocytes, but its regulatory mechanisms need to be further studied. Therefore, we used AA2P, which is a stable derivative of VC that possesses similar biological properties, to explore the mechanism of AA2P to regulate the adipogenesis of 3T3-L1 preadipocytes in vitro.

It has been reported that 200 μM and 250 μM of AA2P could stimulate the lipid accumulation of 3T3-L1 cells and promote the adipogenesis of BMSCs [18, 21]. Consistently, our results showed that AA2P could significantly promote the adipogenesis of 3T3-L1 preadipocytes in a dose-dependent manner (see Additional file 2), and that the concentration of 250 μM is more potent without toxicity to the cells.

We next detected the expression of adipocyte specific factors, such as SREBP-1c, C/EBPβ, C/EBPα and PPARγ. Consistent with a previous study [19], our data showed that AA2P could significantly up-regulate the expression of these factors. However, the expression of SREBP-1c, C/EBPβ and C/EBPα in the AA2P treatment group was consistent with that in the untreated adipogenic group at the late stage of adipogenesis, indicating that AA2P could accelerate the expression of SREBP-1c, C/EBPβ and C/EBPα and maintain the expression of PPARγ. Therefore, the results raise the question of how AA2P accelerates or up-regulates the expression of these factors.

It has been reported that the remodeling of ECM has increased flexibility and changes the connection among collagens, promoting the transcription of adipocyte specific genes, such as C/EBPβ and PPARγ [5]. To determine the roles played by collagens in adipogenesis, we first added EDHB, which is an ideal inhibitor of collagens synthesis. Our data showed that EDHB significantly inhibited the adipogenesis of 3T3-L1 cells by down-regulating the expression of SREBP-1c, C/EBPs and PPARγ.

Collagen VI plays a vital role in the connection among collagens [3]. It was reported that the TG content of BIP is reduced to 50% by EDHB at a concentration of 0.1 mM. Moreover, the ratio of adipose cells was restored to 31.5% when cells that were treated with EDHB were cultured on dishes coated with collagen VI [1], may indicating that collagen VI plays a primary role in adipogenesis. Based on this observation, we focused on the expression of collagen VI. Our data showed that AA2P could significantly up-regulate the expression of collagen VI. Moreover, the mRNA of collagen VI was up-regulated in contrast with the protein of collagen VI after treatment with EDHB. The reason for this is that EDHB only inhibits the assembly and secretion of collagens and does not inhibit the expression of collagen genes, indicating the role of EDHB on collagen synthesis. EDHB significantly inhibited the adipogenesis of 3T3-L1 cells by down-regulating the assembly and secretion of collagen VI to down-regulate the expression of adipocyte specific factors. After treating with AA2P, the expression of collagen VI was up-regulated accompanied by the up-regulation of adipocyte-specific factors, which led to the recovery of the ratio of adipose cells by approximately 50.4%. The reason for this was that EDHB did not completely inhibit the synthesis of collagen VI. AA2P partially promoted the synthesis of collagen and up-regulated the expression of the adipocyte specific factors, resulting in the partially restored adipogenesis of 3T3-L1 cells. Our Western blot results validated the above speculation.

In addition, we only detected collagen VI, not detect other collagens (type I-V), so that we did not know how much remained after adding EDHB (I + EDHB). We specifically knocked down collagen VI, and the ratio of adipose cells was significantly higher than that of the I + EDHB group. Furthermore, the efficiency of AA2P rescuing the ratio of adipose cells in the I + Lenti-ColVI-GFP + AA2P group was significantly higher than that in the I + EDHB + AA2P group. Taken together, we found that other collagens (type I-V) also play a role in adipogenic differentiation, while collagen VI plays a major role in adipogenic differentiation of 3T3-L1 preadipocytes.

Nakajima [1, 2] showed that when confluent bovine intramuscular preadipocytes (BIP) were stimulated with adipogenic medium, there was a significant accretion on the cell surface of the type I-VI collagens, especially type V and VI. When exogenous collagens were supplied to make up for the lack of endogenous products, cultured EDHB-treated cells on type V and VI collagen-coated dishes were the only ones among six collagens to accumulate more TG, with recovery rates of 23.2% and 31.5%, respectively. There was no significant positive or negative effect on TG accumulation in other types of collagens [1]. The above results confirm our previous finding that other collagens (type I-V) also play a role in adipogenic differentiation, while collagen VI plays a major role in adipogenic differentiation.

To further verify the regulation of collagen VI on the adipogenesis of 3T3-L1 cells, we used RNAi to knockdown the α1 subunit of collagen VI, which significantly inhibited adipogenesis of 3T3-L1 cells by down-regulating the expression of SREBP-1c, C/EBPβ, C/EBPα and PPARγ. However, after treating with AA2P, the adipocyte-specific factors and adipogenesis were restored partially. Moreover, the ratio of adipose cells in the Lenti-ColVI-GFP group was higher than that in the EDHB + AA2P group, indicating that AA2P promoted the expression of adipocyte specific factors by up-regulating the synthesis of the collagen VI.

It has been reported that VC could up-regulate the expression of collagen I and III by inhibiting the phosphorylation of ERK1/2 [29, 33–37]. Consistent with previous studies [28, 29], our results indicated that AA2P did not affect the expression of ERK1/2, while it significantly down regulated the ERK1/2 phosphorylation in the early and middle stage of adipogenesis (D0-D7). Moreover, the results showed that the earlier the addition of U0126, the greater the inhibition of adipogenesis (Fig. 5b), further indicating that the ERK signaling plays an important role in the early stage of adipogenesis.

After treating the 3T3-L1 cells with EDHB, the expression trend of the collagen VI was opposite that of the ERK1/2 phosphorylation. In other words, the ERK1/2 phosphorylation was inhibited by the expression of collagen VI. Further studies showed that the ERK1/2 phosphorylation was significantly up-regulated after knockdown of collagen VI. ERK1/2 phosphorylation was decreased after treating with AA2P, while it was significantly higher than that of the Lenti-GFP control group. Bost et al. [38] reported that ERK1 plays a major role in adipogenesis. Consistently, our results showed that knocking down collagen VI mainly impacted ERK1 phosphorylation.

To further verify the role of ERK signaling, we treated the cells with U0126, an inhibitor of ERK1/2 phosphorylation, during 3T3-L1 cells adipogenesis. Previous studies have shown that the ERK1/2 phosphorylation in the early stage of adipogenesis is essential for C/EBPs and PPARγ transcription, but PPARγ is a downstream molecule of ERK1/2 and ERK1/2 phosphorylation could phosphorylate PPARγ. As result reduce in its transcriptional activity, thereby inhibiting adipogenesis [31, 39]. Therefore, the ERK1/2 phosphorylation decreased gradually with adipogenesis.

In this study, we have shown the significantly up-regulated expression of collagen VI after completely inhibiting ERK1/2 phosphorylation by U0126. On the contrary, the adipocyte specific gene factors were significantly down-regulated. This seems to be contrary to our hypothesis. However, AA2P only attenuates ERK1/2 phosphorylation, rather than completely inhibiting it, such as in U0126. In addition, it was surprising that the expression of SREBP-1c was not inhibited by U0126 in the early and middle stage of adipogenesis. However, it was inhibited in the late stage of adipogenesis. This issue needs to be further studied.

Inhibition of ERK1/2 with U0126 results in the inhibition of adipogenesis, which is rescued by AA2P. This is puzzling since the p-ERK1/2 levels tended to decrease with increased adipogenesis.

Previous studies have shown that ERK1/2 phosphorylation in the mitotic clonal expansion stage of adipogenesis is essential for C/EBPβ, while PPARγ is a downstream molecule of ERK1/2, and the activation of ERK1/2 could phosphorylate PPARγ; therefore, the p-ERK1/2 levels tended to decrease with increased adipogenesis [31, 39]. The present data showed that ERK1/2 phosphorylation mainly regulated the adipogenesis of 3T3-L1 cells in the early and middle stage of adipogenesis, especially in the early stage of adipogenesis (Fig. 5b).

Our results showed that AA2P promotes the adipogenesis of the 3T3-L1 cells by attenuating ERK signaling to up-regulate collagen VI. In the absence of p-ERK1/2, AA2P could still partially rescue the inhibitory effect of U1026 on the adipogenic differentiation of 3T3-L1 preadipocytes. The possible reason for this is that AA2P could promote the adipogenic differentiation of preadipocytes by other pathways.

VC has an important epigenetic regulatory function. Yin et al. [40] have shown that VC could enhance the ten-eleven translocation (Tet)-mediated oxidation of 5-methylcytosine to 5-hydroxymethylcytosine and promote the demethylation of DNA, thereby activating gene expression. Further studies [41] have shown that PPARγ could guide the local demethylation of its binding site (PPAR response elements, PPREs) and activate the genes involved in adipogenesis (such as perilipin), promoting the adipogenic differentiation of 3T3-L1 preadipocytes. It could be speculated that AA2P also promotes the adipogenic differentiation of 3T3-L1 preadipocytes by promoting the expression of PPARγ and acting as a cofactor of Tet to promote the expression of the genes involved in adipogenesis. However, the role of this pathway in promoting the adipogenic differentiation of 3T3-L1 preadipocytes is relatively weak. Therefore, AA2P promotes the adipogenesis of 3T3-L1 cells by mainly attenuating ERK signaling to up-regulate collagen VI.

## Conclusions

**Fig. 6:**
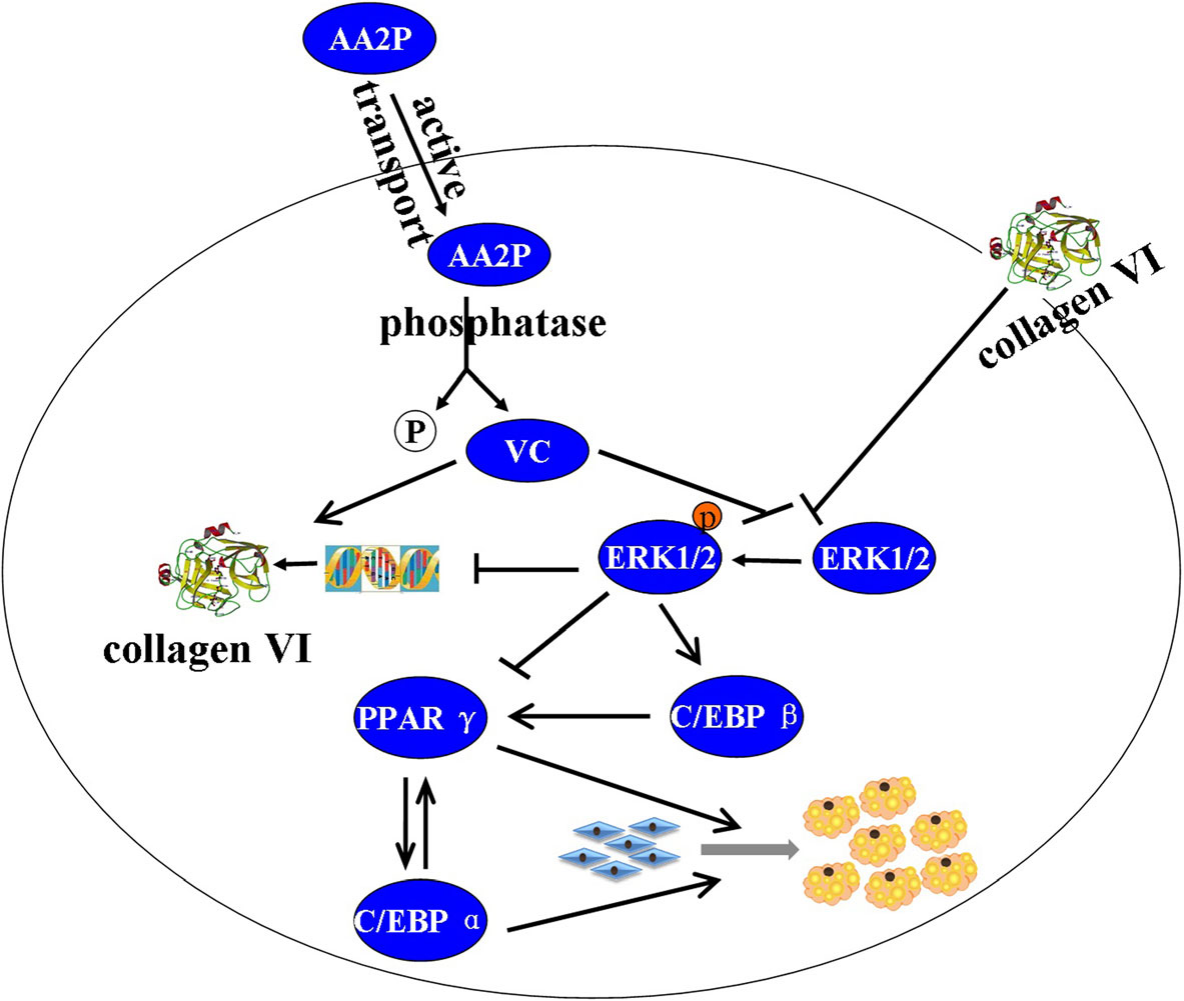
The possible mechanism of AA2P promoting the adipogenesis of 3T3-L1 cells. AA2P enters the cells by active transport and plays two roles. First, AA2P promotes the assembly and secretion of collagen VI as the hydroxyl factor co-enzyme, and then down-regulates the phosphorylation of ERK1/2. Second, VC down regulates ERK1/2 phosphorylation directly by a certain mechanism

Our data indicated that AA2P promotes 3T3-L1 preadipocyte adipogenic differentiation. The speculative mechanism of effects of AA2P on cell adipogenesis is as follows: VC promoted the synthesis and secretion of collagen VI as the hydroxyl factor co-enzyme and down-regulated the ERK1/2 phosphorylation to promote the adipogenesis of the preadipocytes by up-regulating the expression of adipocyte-specific factors, such as SREBP-1c, C/EBPβ, PPARγ and C/EBPα; AA2P up-regulated the expression of collagen VI by attenuating ERK1/2 phosphorylation, further up-regulating adipocyte-specific factors, and finally promoting the adipogenesis of 3T3-L1 cells (Fig. 6). It is noteworthy that VC accelerated the lipid accumulation on 3T3-L1 cells, at least in part by attenuating the ERK1/2 phosphorylation to up-regulate collagen VI. Further work need to be down carefully whether VC plays similar role in vivo, since it was reported that VC has dual function in adipogenesis in vivo.

## Additional files

Additional file 1: Screened the MOI value of the lentivirus and detected the knockdown efficiency of Col VI: **a** 5 × 104 3T3-L1 preadipocytes/ml were plated in 12-well plates overnight and infected with Lenti-ColVI-GFP lentivirus by addition of lentivirus diluted into 0.5 ml of Dulbecco’s modified eagle medium (DMEM) containing 5% NBCS and polybrene at the MOI of 0, 20, 50 and 100 for 12 h at 37 °C. The medium was then replaced with growth medium until it reached 100% confluence. Adipogenic induction was initiated after 48 h of confluence. The controls were infected with NC lentivirus (I + Lenti-GFP); **b** After screening out the optimal MOI value of the lentivirus, we detected the knockdown efficiency of Col VI using real-time quantitative reverse transcription. Values are expressed as the mean ± SEM. **P < 0.01 versus the control group (*n* = 3). (PDF 234 kb)

Additional file 2: The effect of AA2P on adipogenesis is dose-dependent: a 3T3-L1 preadipocytes were seeded on 12-well plate with growth medium (DMEM, 10% new-born calf serum, penicillin (100 U/ml), streptomycin (100 mg/mL) and 2 mM L-glutamine) to let the cells reached 100% confluent. Two days later, the cells were induced for mature adipocytes with cocktail adipogenic induction medium (growth medium supplemented with 1.7 μM insulin) for an additional 2 days. The medium was finally replaced with growth medium for 10 more days. AA2P was added from the D0 to D14 at final concentrations of 0, 100, 250 and 500 μM, respectively; b On day 14, the cells were stained with Oil red O; The lipid the staining was quantified by extracting the dye with 100% isopropanol and measuring the absorbance at 520 nm. Values are expressed as the mean ± SEM. *P < 0.05 versus the control group (*n* = 3). (PDF 1599 kb)

## Abbreviations

AA2P: L-ascorbic acid-2-phosphate
BIP: Bovine intramuscular preadipocytes cell line
ColVI: Collagen VI
DMSO: Dimethyl sulfoxidein
ECM: Extracellular matrix
EDHB: Ethyl-3, 4-dihydroxybenzoate
ERK: Extracellular signal-regulated kinase
GFP: Green fluorescent protein
I: Induction
MAPK: Mitogen-activated protein kinase
N.I.: Non-induction
TG: Triglyceride
VC: Vitamin C
