# Supplementary material for: Ascorbic acid promotes 3T3-L1 cells adipogenesis by attenuating ERK signaling to upregulate the collagen VI
Source: Nutr Metab (Lond). 2017 Dec 28;14:79. doi: 10.1186/s12986-017-0234-y (PMC5745638; doi:10.1186/s12986-017-0234-y)
Supplement: Supplementary file 1 — Screened the MOI value of the lentivirus and detected the knockdown efficiency of Col VI: a 5 × 10^4^ 3T3-L1 preadipocytes/ml were plated in 12-well plates overnight and infected with Lenti-ColVI-GFP lentivirus by addition of lentivirus diluted into 0.5 ml of Dulbecco’s modified eagle medium (DMEM) containing 5% NBCS and polybrene at the MOI of 0, 20, 50 and 100 for 12 h at 37 °C. The medium was then replaced with growth medium until it reached 100% confluence. Adipogenic induction was initiated after 48 h of confluence. The controls were infected with NC lentivirus (I + Lenti-GFP); b After screening out the optimal MOI value of the lentivirus, we detected the knockdown efficiency of Col VI using real-time quantitative reverse transcription. Values are expressed as the mean ± SEM. **P < 0.01 versus the control group (n = 3). (PDF 234 kb) [file 12986_2017_234_MOESM1_ESM.pdf]

Additional file 1

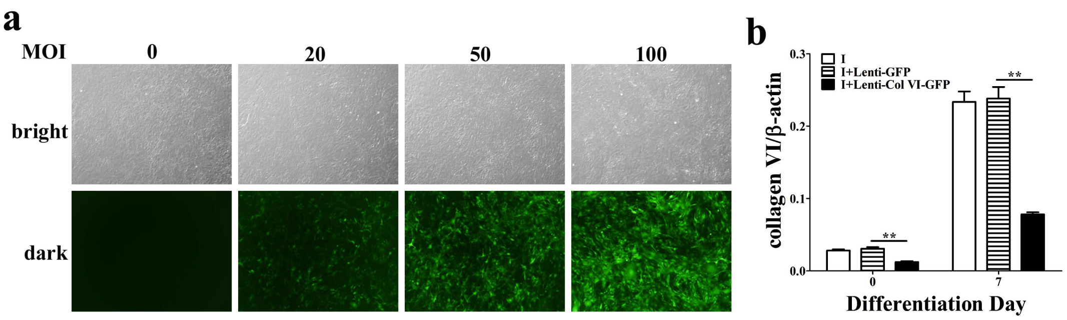

Screened the MOI value of the lentivirus and detected the knockdown efficiency of Col VI: **a** 5 × 10^4 3T3-L1 preadipocytes/ml were plated in 12-well plates overnight and infected with Lenti-ColVI-GFP lentivirus by addition of lentivirus diluted into 0.5 ml of Dulbecco's modified eagle medium (DMEM) containing 5% NBCS and polybrene at the MOI of 0, 20, 50 and 100 for 12 hours at 37°C. The medium was then replaced with growth medium until it reached 100% confluence. Adipogenic induction was initiated after 48 hours of confluence. The controls were infected with NC lentivirus (I + Lenti-GFP); **b** After screening out the optimal MOI value of the lentivirus, we detected the knockdown efficiency of Col VI using real-time quantitative reverse transcription. Values are expressed as the mean ± SEM. $^{**}P < 0.01$ versus the control group (n = 3).
