# Supplementary material for: Ascorbic acid promotes 3T3-L1 cells adipogenesis by attenuating ERK signaling to upregulate the collagen VI
Source: Nutr Metab (Lond). 2017 Dec 28;14:79. doi: 10.1186/s12986-017-0234-y (PMC5745638; doi:10.1186/s12986-017-0234-y)
Supplement: Supplementary file 2 — The effect of AA2P on adipogenesis is dose-dependent: a 3T3-L1 preadipocytes were seeded on 12-well plate with growth medium (DMEM, 10% new-born calf serum, penicillin (100 U/ml), streptomycin (100 mg/mL) and 2 mM L-glutamine) to let the cells reached 100% confluent. Two days later, the cells were induced for mature adipocytes with cocktail adipogenic induction medium (growth medium supplemented with 1.7 μM insulin) for an additional 2 days. The medium was finally replaced with growth medium for 10 more days. AA2P was added from the D0 to D14 at final concentrations of 0, 100, 250 and 500 μM, respectively; b On day 14, the cells were stained with Oil red O; The lipid the staining was quantified by extracting the dye with 100% isopropanol and measuring the absorbance at 520 nm. Values are expressed as the mean ± SEM. *P < 0.05 versus the control group (n = 3). (PDF 1599 kb) [file 12986_2017_234_MOESM2_ESM.pdf]

# Additional file 2

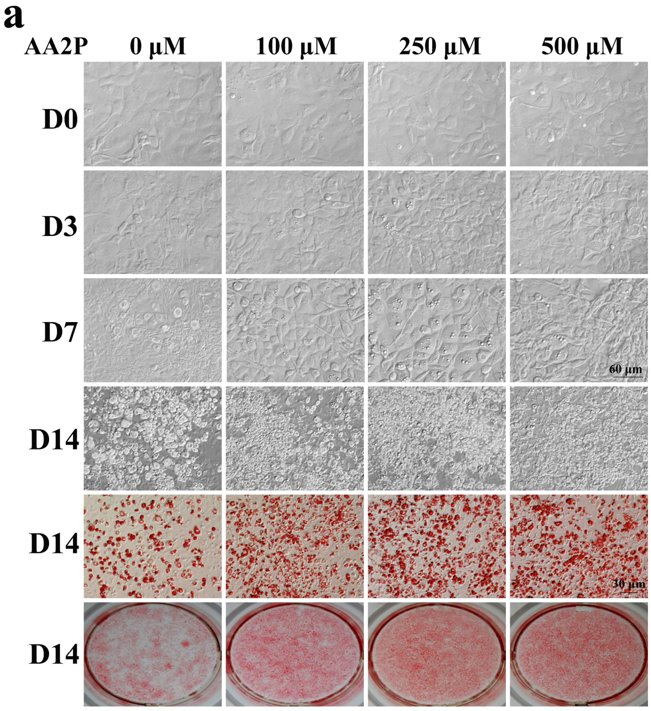


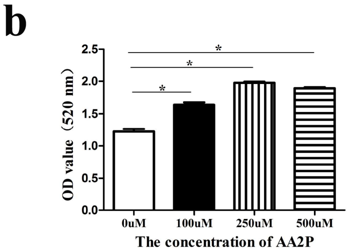


The effect of AA2P on adipogenesis is dose-dependent: **a** 3T3-L1 preadipocytes were seeded on 12-well plate with growth medium (DMEM, 10% new-born calf serum, penicillin (100 U/ml), streptomycin (100 mg/mL) and 2 mM L-glutamine) to let the cells reached 100% confluent. Two days later, the cells were induced for mature adipocytes with cocktail adipogenic induction medium (growth medium supplemented with 1.7 µM insulin) for an additional 2 days. The medium was finally replaced with growth medium for 10 more days. AA2P was added from the D0 to D14 at final concentrations of 0, 100, 250 and 500 µM, respectively; **b** On day 14, the cells were stained with Oil red O; The lipid the staining was quantified by extracting the dye with 100% isopropanol and measuring the absorbance at 520 nm. Values are expressed as the mean ± SEM. **P* < 0.05 versus the control group (n = 3).
